# Examining Quitting Experiences on Quit Vaping Subreddits From 2015 to 2021: Content Analysis

**DOI:** 10.2196/52129

**Published:** 2024-10-25

**Authors:** Elexis Kierstead, Nathan Silver, Michael Amato

**Affiliations:** 1 Truth Initiative Washington, DC United States

**Keywords:** quitting vaping, social media, tobacco policy, cessation, e-cigarette, electronic cigarette, smoking, vaping, cessation programs, social support, peer support

## Abstract

**Background:**

Despite the prevalence of vaping nicotine, most nicotine cessation research remains focused on smoking cigarettes. However, the lived experience of quitting smoking is different from quitting vaping. As a result, research examining the unique experiences of those quitting vaping can better inform quitting resources and cessation programs specific to e-cigarette use. Examining Reddit forums (ie, subreddits) dedicated to the topics of quitting vaping nicotine can provide insight into the discussion around experiences on quitting vaping. Prior literature examining limited discussions around quitting vaping on Reddit has identified the sharing of barriers and facilitators for quitting, but more research is needed to investigate the content comprehensively across all subreddits.

**Objective:**

The objective of this study is to examine content across quit vaping subreddits since their inception to better understand quitting vaping within the context of the expanding nicotine market.

**Methods:**

All posts from January 2015 to October 2021 were scraped from all quit vaping subreddits: r/QuittingJuul, r/QuitVaping, r/quit_vaping, and r/stopvaping (N=7110). Rolling weekly average post volume was calculated. A codebook informed by a latent Dirichlet allocation topic model was developed to characterize themes in a subsample of 695 randomly selected posts. Frequencies and percentages of posts containing each coded theme were assessed along with the number of upvotes and comments.

**Results:**

Post volume increased across all subreddits over time, spiking from August – September of 2019 when vaping lung injury emerged. Just over 52% of posts discussed seeking social support and 16.83% discussed providing social support. Posts providing support received the most positive engagements (i.e. upvotes) of all coded categories. Posts also discussed physical and psychological symptoms of withdrawal (30.65% and 18.85%, respectively), strategies for quitting including: quitting cold turkey (38.33%), using alternative nicotine products (17%), and tapering down nicotine content (10.50%). Most posts shared a personal narrative (92.37%) and some discussed quit motivation (28.20%) and relapse (14.99%).

**Conclusions:**

This work identifies a desire for peer-to-peer support for quitting vaping, which reinforces existing literature and highlights characteristics of quitting vaping specific to a changing nicotine product environment. Given that posts providing social support were the most upvoted, this suggests that subreddit contributors are seeking support from their peers when discussing quitting vaping. Additionally, this analysis shows the sharing of barriers and facilitators for quitting, supporting findings from prior exploration of quit vaping subreddits. Finally, quitting vaping in an ever-growing nicotine market has led to the evolution of vaping-specific quit methods such as tapering down nicotine content. These findings have direct implications for quit vaping product implementation and development.

## Introduction

The nicotine product landscape, which used to be dominated almost exclusively by cigarettes, has expanded over the last decade to include new noncombustible products varying in nicotine delivery method and product characteristics [1,2]. Nicotine vapes, that is, e-cigarettes, are currently the most popular tobacco product used by US youth and young adults and have increased in popularity among adult users [1-3]. The drastic increase in vaping nicotine is accompanied by a corresponding increase in an interest to quit [4]. While nicotine vaping cessation shares many similarities with cigarette smoking cessation, it also differs in important ways, including variability of products, social acceptability, place-based restrictions of product use, and reasons for quitting [5,6]. As the e-cigarette and nicotine product markets continue to rapidly evolve, it is critical to continue monitoring the evolving nature of cessation from these products to ensure that interventions and treatment programs are effective at providing users with the support they need.

Reddit is a highly trafficked forum-based social media site used by just below 20% of US adults [7], wherein users can post on a discussion forum about a particular topic, that is, subreddit, and other users can comment, upvote, or downvote the post. Upvotes and downvotes indicate agreement and support or disagreement and dislike, respectively, and determine the likelihood that content will appear in a user’s feed. Reddit users are more likely to be male, young adults, and White [8]. Reddit provides a unique source of information, granting insight into lived experiences without external researcher intervention and observation [9-11]. Examining content on subreddits—user-run forums organized around a topic or interest group—specific to quitting vaping nicotine provides insight into individual experiences within the context of the expanding tobacco product market. Additionally, prior literature has established that social media sites, such as Reddit, can provide social support to users [12-14], and social support on social media has even been tied to increases in self-efficacy to quit tobacco [15]. Reddit’s utility as a discussion-based forum and social media site that individuals may turn to for social support emphasizes the importance of understanding if and how those trying to quit vaping are using this platform.

The changing nicotine product landscape has resulted in the availability of a wide variety of nicotine products for which quitting experiences may differ. The rise of JUUL products led to the widespread popularity of vape products, peaking in 2018 when the US Food and Drug Administration (FDA) characterized youth vape use as an “epidemic” [16]. Tobacco policy developments responding to this epidemic resulted in shifts in the nicotine product market. After the outbreak of lung injury cases initially connected to nicotine vape products [17], the FDA restricted the sale of flavored pod-based nicotine vapes [16]. As a result, disposable flavored nicotine vapes not restricted by this policy rose to popularity, leading to further proliferation of new vaping products and brands [3,18,19]. The FDA also required all tobacco products that emerged to submit a premarket tobacco product application (PMTA) by September 9, 2021 [20]. Through this process, the FDA reviews the scientific evidence for risks and benefits to public health for each product and determines if they are permitted to market their products in the United States. If denied authorization to market their products, said products could be removed from the US market. PMTA review is an important regulatory process implemented to address the presence of previously unregulated e-cigarette products that continue to be sold broadly on the US market [21]. Following the regulatory scrutiny surrounding e-cigarettes, recent years have seen the emergence of other noncombustible nicotine products—such as Zyn and On!—gaining popularity on the marketplace, especially among current tobacco users [22-24]. Further examination of the experiences of those quitting vaping nicotine within the context of this ever-changing market is needed.

The experiences of quitting vaping nicotine and quitting smoking are different [25,26]. Unlike quitting smoking, those quitting vaping must overcome the appeal of flavors, the inherent convenience and discreteness of the product, and a reported lack of self-awareness of how often one is using the product [6]. Moreover, those quitting vaping report a lack of trusted information on product safety and greater social acceptability of product use as unique challenges compared to quitting cigarettes [6]. Further investigation into the nuances of the quitting experience for vape product users is needed to better inform quitting interventions.

Reddit, as an online discussion-based social media platform, provides candid insight about the quitting process, as this public discourse is conducted outside of a research setting. Reddit has previously been used as a data source to examine quitting vaping [27-29]. One study examined posts from the r/QuitVaping subreddit over a 4-week period in 2020, qualitatively evaluating the barriers and facilitators to use. This work identified withdrawal symptoms and the intensity of addiction as the biggest barriers, and distracting oneself and maintaining a positive mindset as the most effective facilitators [29]. In light of useful insights gained from previous examinations of the quitting experience on Reddit, there is value in expanding the scope of inquiry to longitudinally examine the growth of multiple online quitting communities on the platform, as well as the most common topics of conversation.

We examine all posts from all subreddits related to quitting vaping, encompassing the entire lifetime of these forums, thus allowing us to contextualize our findings within a developing marketplace, a unique contribution to the literature. We conducted a content analysis to summarize themes present in the posts using an inductively developed codebook. Through this work, we asked (1) How had the volume of use of quit vaping subreddits changed across time? and (2) What were the most common topics of conversation on these forums?

## Methods

### Procedure

To comprehensively examine how those trying to quit vaping use Reddit, we analyzed all posts on subreddits dedicated to quitting vaping nicotine. Post content, number of comments, and upvotes were used to examine broad trends, among other metadata. Moreover, we used the *Gensim* package, developed by RARE Technologies Ltd, for Python (version 3.10; Python Software Foundation) to conduct an unsupervised machine learning analysis (latent Dirichlet allocation [LDA] topic modeling) of all posts to develop a codebook for human-coded content analysis on a random sample of posts. Descriptive analysis of user engagement with Reddit posts, including comments and upvotes (a platform feature used to crowdsource evaluations of the relevance of a post from forum members), was aggregated for each coded feature.

### Sample and Measures

Using the Pushshift application programming interface, all posts (N=7110) and comments (N=41,019) were scraped from 4 quit vaping subreddits: r/QuittingJuul, r/QuitVaping, r/quit_vaping, and r/stopvaping. The posts spanned from January 12, 2015, the first post in r/quit_vaping, to October 20, 2021, following the PMTA deadline in September of 2021 and other seismic shifts in the nicotine product marketplace. Post text, timestamp, subreddit of origin, post author, number of upvotes, and number of comments were collected for each post.

### Codebook Development

First, rolling weekly averages of post volume over time within and across all 4 subreddits were calculated. Second, post and comment totals were aggregated at the user level. Next, we developed a codebook to characterize themes across all Reddit posts. Whereas previous research focused on a specific time interval or subreddit [27-29], we examine the full corpus of texts (7110 posts) on all quit-related subreddits from their inception. Given this volume of texts, we used an inductive, data-driven method—LDA topic modeling—to perform an initial “first pass” through the data to identify common topics. In contrast to theory-driven approaches to content analysis, this inductive approach can help identify common topics of discussion on quitting-related subreddits that may not be accounted for by existing theory [30,31]. LDA is a dimension reduction procedure analogous to confirmatory factor analysis wherein a corpus of texts is stripped of syntactical structure, including the removal of *stop words* like “and,” “or,” and “but”; *stemming* to remove suffixes (eg, “ed” and “ing”); *tokenizing* to make each unique word a variable; and *vectorization* to create a sparse matrix that essentially dummy codes the presence or absence of every possible word in each individual text [32]. The LDA algorithm then identifies latent topics based on words that both co-occur and help to differentiate between topics. We specify the number of topics a priori based on a perplexity graph, which visualizes model fit as a function of the optimal number of topics. In our case, a 12-topic model had the highest perplexity [33]. LDA then produces probabilistic factor loadings for each latent topic indicative of the likelihood that a given text shares a latent topic with other texts based on the weighted values of the words in that text. Consistent with convention [34], we set a coherence threshold of 0.70 to identify the most highly weighted words and example texts with high factor loadings for each latent topic that we used to interpret the topic model (Table 1).

**Table 1:** Latent Dirichlet allocation (LDA) topic model–determined themes to inform content analysis from a sample of 7110 posts on quit vaping subreddits from January 2015 to October 2021.

Topic number	Topic name	Relevant words	Example
1	Asking for help	need, hard, stop, trying	"Please help I can't quit: I've been vaping for 4 years now and I seriously wish I never started. I've tried to quit but I can't. I don't even get buzzed anymore. How did everyone else quit? I've been thinking of buying a CBD vape or something with a low percent nic to slowly get off, would this help?"
2	Initiation and progression	hard, stop, trying, gum, habit, addiction, hitting	"I never smoked cigarettes before but in the height of the Juul era several years ago all of my friends switched to vaping. I picked up the habit."
3	Sleeplessness and brain fog	ago, never, thought, felt, else, worse, sleep, wake, brain	"Like I feel like I'm not a person like I don't exist. Like I don't want to do anything like the only thing I did today was browse my computer. I just have been laying on my bed because I feel so useless and tired. I can't focus for the life of me."
4	Quit aids	using, juice, nic, gum, cigarettes, symptoms, CBD	"Mine: lollipops, fidget cube, Bubly water, those QuitGo fake cigarettes, Nicolette lozenges. Still looking for more, I'm on day 6 What are your coping mechanisms?"
5	Quit motivation	work, people, health, news, long, life	"I'm 17 and I quit vaping because my 10 month old niece is moving in with me on the 15th and I want to be better for her and I'd like to be completely nicotine free before she lives here."
6	Consumption method	salts, pod, used, around, juice, vs, mg/ml	"GUIDE: Easy weaning off JUUL w/ diminishing nicotine salt strengths (w/ links): If you JUUL you may have noticed how absolutely ass-kickingly strong the nicotine salt solution in JUUL is compared to analogs and non-nicotine salt vaping. You may have switched from JUUL 5% to the new 3% pods and found them still too strong. JUUL doesn't offer anything below 3%."
7	Anxiety, respiratory or cardiovascular	chest, pain, heart, breath, shortness, attack, panic, tightness	"It's day 17 and I am having 2-3 panic attacks a day and dealing with depression. All of these symptoms onset the day I quit. Is this normal? And how long until it starts to go away"
8	Study recruitment	study, link, survey, research, based, users, mobile	"If you use **e-cigarettes or vape** and are **16-20 years old**, you may qualify for a **remote research study** with the Medical University of South Carolina."
9	Relapse circumstances	relapsed, drunk, cigars, spliffs, scare	"I'm just going to go back to college where all of my friends have nicotine and there's no way I'm going to say no when they offer it to me, especially when drunk."
10	Weight gain	weight, gain, hungry	"I started vaping 3mg about 3 years ago because I learned that nicotine was an appetite suppressant, and that sounded perfect to my (at the time) anorexic brain."
11	Self-help or journey metaphor or guidance	allen, carr, book	"Any advice is appreciated and explaining what to expect in the coming days will be very helpful to my journey"
12	Relational problems while quitting	freebase, husband, refused, snappy, smashed	"My partner who I live with still vapes regularly. So when I decided to try to quit I knew this would be a challenge, as having it around, in sight all the time would be hard."

LDA also has significant limitations, leading us to prefer not to rely on topic modeling alone. First, perplexity is an imprecise measure with the identified topics indicative of patterns of shared words that may or may not be interpretable. As a result, some “topics” identified by the model may not be topics in reality. Most importantly, although LDA is an effective and efficient way to sift through large amounts of text data for broader trends, the accuracy of any given factor loading is limited. While we can confidently say that a topic identified by the model is prevalent across the broader corpus of text, a given text that loads high on a latent topic has a relatively low likelihood of being about that topic. However, the inductive generation of topics proved useful in developing a codebook to exhaustively represent the data.

The topic model identified 12 latent topics that were refined by the authors to be human coded on a random sample of 695 posts. Based on established conventions for media-based content analyses [35], we reserved 10% (n=69) of our sample for establishing reliability. Two trained research assistants coded the reserved sample, resolving discrepancies via discussion until intercoder reliability for each coded category exceeded a minimum Cohen κ threshold of 0.70 [35]. Coded categories with low reliability were dropped from further analysis, resulting in 6 major themes, 3 of which contained subthemes, for a total of 13 codes. Codes were not mutually exclusive, allowing for multiple codes in the same post. Table 2 provides a full accounting of our codebook, criteria, and reliability information.

**Table 2:** Expert-determined, topic model–informed codebook for examining themes among a random sample of 695 posts across all quit vaping subreddits from January 2015 to October 2021 with reliability between trained coders.

Code	Definition	Example	Cohen κ
Social support	Text mentions or contains users interacting with one another in a socially supportive way, seeking or providing peer support, or mentioning social support in their vaping cessation efforts	“I believe in you!”	0.91
Seeking (SS^a^)	Text contains asking or searching for support or advice from other Reddit users	“throw me your strategies for not juuling at parties”	0.91
Providing (SS)	Text contains an offer for advice or explicitly gives social support to other Reddit users, either with a clear recipient or to the general userbase	“Here's a few helpful tips”	0.84
Withdrawal	Text mentions withdrawal from vaping products, either in apprehension or as lived experience	“2 weeks! The only cravings now are psychological- when I get bored, or am in situations where I used to Juul heavily (like playing video games)”	0.88
Physical (W^b^)	Text mentions physical symptoms of withdrawal from vaping products, including cravings, appetite changes, weight gain or loss, respiratory symptoms, cardiovascular symptoms, flu-like symptoms (ie, aches and pains, runny nose), nausea, cosmetic appearance (ie, skin or acne or bags under eyes)	“Overall, definitely feeling much better physically. Way less dehydrated, happier, sleeping better and feeling more energetic in the am.”	0.86
Psychological (W)	Text mentions emotional or mental symptoms of withdrawal from vaping products, including anxiety or depression, brain fog or confusion, anger, relationship struggles, confusion between emotional and physical symptoms	“I feel amazing! The first 3 days were awful - mostly mental symptoms; irritable, tired, increased appetite, headache, extreme cravings.”	0.87
Quit strategies	Text mentions strategies used to quit using vaping products, which can include seeking or providing guidance or life experience	“For the craving and withdrawals all I did was drink ice cold water and play basketball outside in my backyard after work to keep myself busy .”	0.97
Cold turkey (QS^c^)	Text mentions completely ceasing use of tobacco or nicotine products at one point in time as a strategy for quitting vaping	“I quit 25 mg nic salts cold turkey about 5 days and 2 hours ago.”	1
Tapering (QS)	Text mentions the slow reduction of nicotine content in e-liquid in an effort to quit or the slow reduction in use in a vaping product over time. Can also include tapering regarding frequency of use of nicotine products, regardless of change in e-liquid content (eg, individual using juul 5 days a week, tapering down to 3 days a week, and so on)	“I had previously dropped from 50 mg to 25 mg as the first step of my quitting journey.”	1
Alternative products (QS)	Text mentions the use of alternate tobacco or nicotine products as a quit strategy (eg, nicotine gum, nicotine pouches, cigarettes, cannabis products, non-nicotine vaping devices, etc.) This includes FDA^d^-approved nicotine replacement therapy cessation products.	“Im chewing gum now but if im honest its not helping as much as it should”	0.87
Personal narrative	Text contains a personal story on the forum often containing lived experiences to either ask for or give guidance and support	“So I haven't been using JUUL exclusively for the past year since I started using electronic cigs, vapes, etc.”	0.88
Quit motivation	Text contains mention of sources of motivation to quit vaping or a personal assessment of how motivated they feel to quit. This sentiment can either be positive or negative, discussing why they are or aren't motivated to quit	“Those thoughts kept me motivated my first three days”	0.97
Relapse	Text mentions beginning to vape again after intending to quit using the product, or after a period of sustained abstinence from vaping, either as a lived experience or in apprehension	“Trying to quit but keep slipping up”	1

^a^SS: social support.

^b^W: withdrawal.

^c^QS: quitting strategy.

^d^FDA: Food and Drug Administration.

### Statistical Analyses

Frequencies and percentages of posts containing each coded theme were tallied along with measures of central tendency for the number of upvotes and comments within each coded theme. The number of comments was counted for each post; however, these comments were not analyzed for content.

### Ethical Considerations

Study data were collected from a publicly available data source, Reddit; therefore, no ethics approval was required. The data were anonymized for analysis and publication.

## Results

### Post Volume Over Time

**Figure 1:**
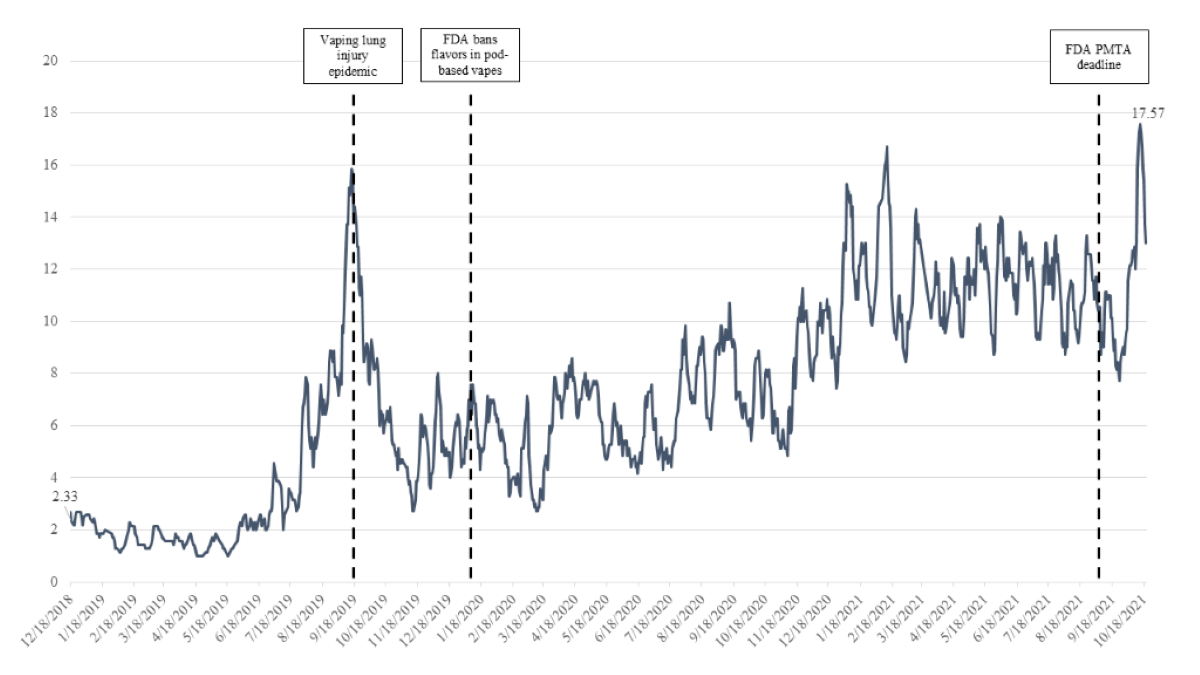
Rolling weekly average posts aggregated across 4 quit vaping subreddits from January 2015 to October 2021 (N=7110). FDA: Food and Drug Administration; PMTA: premarket tobacco product application.

**Figure 2:**
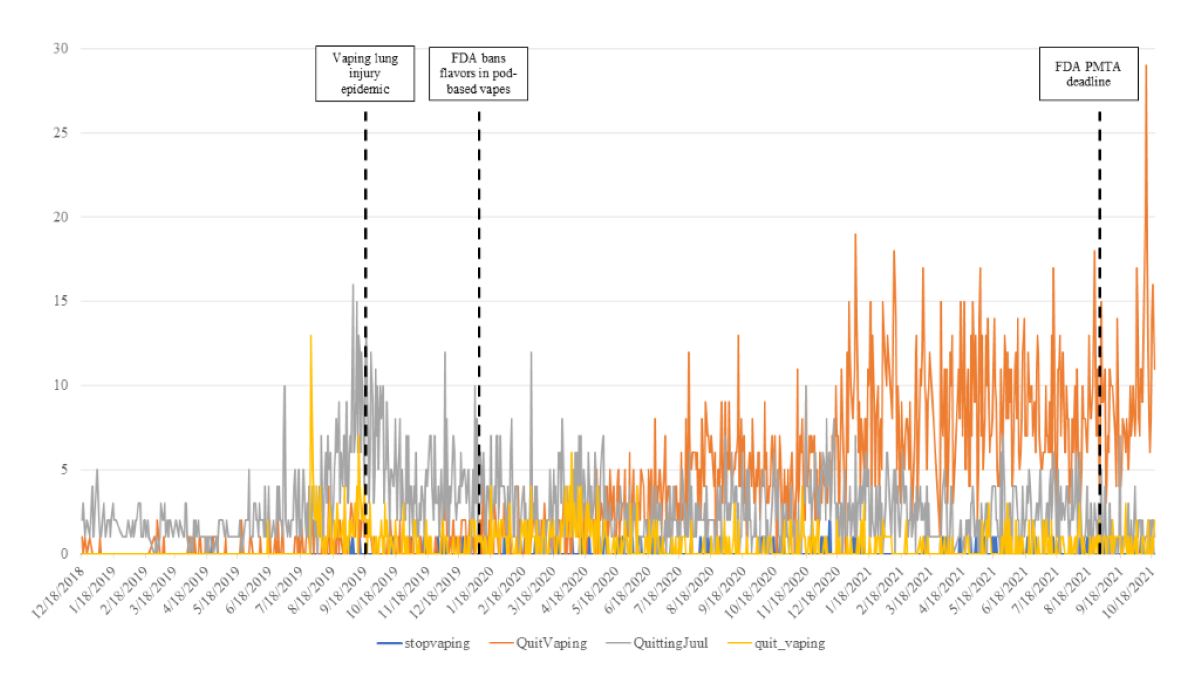
Post volume across quit vaping subreddits from January 2015 to October 2021 (N=7110). FDA: Food and Drug Administration; PMTA: premarket tobacco product application.

Across all 4 subreddits, rolling weekly average posts showed that overall use of these subreddits had grown over time. After an initial peak in overall post volume from August to September 2019, posts trended upward over time (Figure 1). When examining post volume by subreddit, we identified that r/QuittingJuul was initially the most frequently used subreddit, until May to June 2020, when r/QuitVaping overtook r/QuittingJuul in post volume. The other 2 subreddits, r/quit_vaping and r/stopvaping, were infrequently used (Figure 2).

### User Contribution Characteristics

The sample consisted of 7110 total posts by 3197 unique users. Notably, 2336 posts were not affiliated with an author name either because the associated account was deleted or the user requested that Pushshift delete their data. Most users posted infrequently (median 1, IQR 1-1), with only 23.8% (761/3197) of users posting more than once and 1.9% (60/3197) of users posting more than 5 times. The posts garnered 41,019 comments (median 4, IQR 2-6 comments per post). A total of 7873 unique users posted a median of 2 (IQR 1-4) comments, each with 23.9% (n=1882) of users posting 5 or more comments and 9.7% (n=764) posting 10 or more comments. However, 12 users each posted more than 100 comments. Also, 5396 comments were not associated with a username, suggesting there may have been more users commenting than those we documented.

### Content Analysis

Table 3 summarizes all 13 codes, including the frequency at which they appeared in the sample, as well as median upvotes and median comments.

**Table 3:** Frequency and overall percent of coded sample of 695 quit vaping–related subreddit posts from January 2015 to October 2021 containing each theme from a topic-model derived, expert-determined codebook.

Code	Frequency, n (%)	Upvotes, median (IQR)	Comments, median (IQR)
Social support	467 (67.19)	5 (3-10)	4 (2-6)
Seeking (SS^a^)	362 (52.09)	4 (2-7)	4 (2-7)
Providing (SS)	117 (16.83)	15 (8-22)	4 (2-6)
Withdrawal	291 (41.87)	6 (3-11)	4 (2-6)
Physical (W^b^)	213 (30.65)	6 (3-12)	4 (2-6)
Psychological (W)	131 (18.85)	7 (3-13)	4 (2-6)
Quit strategies	389 (55.97)	6 (3-13)	4 (2-6)
Cold turkey (QS^c^)	266 (38.33)	7 (3-13)	4 (2-6)
Tapering (QS)	73 (10.50)	6 (3-10)	4 (2-6)
Alternative products (QS)	118 (17)	5 (3-11)	4 (2-6)
Personal narrative	642 (92.37)	6 (3-13)	4 (2-6)
Quit motivation	196 (28.2)	7 (3-13)	4 (2-6)
Relapse	104 (14.99)	4 (3-9)	4 (2-5.5)

^a^SS: social support.

^b^W: withdrawal.

^c^QS: quitting strategy.

#### Social Support

Over two-thirds of posts contained mentions of *social support* (467/695, 67.19%). We found that 52.09% (362/695) of posts in the sample were *seeking social support*, making up 77.52% (362/467) of the posts coded as *social support*. Meanwhile, 16.83% (117/695) of the total coded posts and 25.05% (117/467) of the *social support* posts *provided social support*. Posts *seeking social support* received a median of 4 (IQR 2-7) upvotes and 4 (IQR 2-7) comments, while posts *providing social support* received a median of 15 (IQR 8-22) upvotes and 4 (IQR 2-6) comments.

#### Withdrawal

We found 41.87% (291/695) of posts discussed *withdrawal* from vaping nicotine. Of the total coded sample, 30.65% (213/695) of posts discussed *physical withdrawal* symptoms, making up 73.2% (213/291) of posts mentioning withdrawal. To a lesser extent, 18.85% (131/695) of the total sample and 45.02% (131/291) of *withdrawal* posts discussed *psychological withdrawal* symptoms. Examples of physical withdrawal symptoms include cravings, heart palpitations, mouth pain, nausea, or changes to appetite, while psychological symptoms of withdrawal include increased anxiety, changes to mood, or brain fog. Mentions of *physical withdrawal* received a median of 6 (IQR 3-12) upvotes and 4 (IQR 2-6) comments, while *psychological withdrawal* mentions received a median of 7 (IQR 3-13) upvotes and 4 (IQR 2-6) comments.

#### Quit Strategies

We found 55.97% (389/695) of posts discussed *strategies for quitting*. Of the total sample, 38.33% (266/695) of posts discussed *quitting cold turkey*, making up 68.38% (266/389) of the posts mentioning *quit strategies*. About 10.50% (73/695) of the full coded sample and 18.77% (73/389) of the *strategies for quitting* posts discussed *tapering down nicotine content* as a method of quitting. Approximately 17.00% (118/695) of the full sample and 30.33% (118/389) of the *strategies for quitting* posts mentioned *using alternative products* to quit. Notably, post authors reported use of both FDA-approved and unapproved quit aids ranging from nicotine replacement therapy (NRT) to recreational oral nicotine products (eg, Zyn). Posts discussing *quitting cold turkey* received a median of 7 (IQR 3-13) upvotes and 4 (IQR 2-6) comments, posts discussing *tapering down* received a median of 6 (IQR 3-10) upvotes and 4 (IQR 2-6) comments, and posts discussing *alternative products* received a median of 5 (IQR 3-11) upvotes and 4 (IQR 2-6) comments.

#### Personal Narratives, Quit Motivation, and Relapse

The majority of posts in the sample (642/695, 92.37%) contained *personal narratives* drawing on and sharing experiences of the post author in the text. These posts received a median of 6 (IQR 3-13) upvotes and 4 (IQR 2-6) comments.

We found 28.2% (196/695) of the sample contained discussion around *motivation to quit* vaping. Themes around health and wellness were commonly found as motivating reasons for quitting vaping. Posts discussing *quit motivation* received a median of 7 (IQR 3-13) upvotes and 4 (IQR 2-6) comments.

We found that 14.99% (104/695) of posts mentioned *relapsing* vaping after a period of cessation. These posts mentioning *relapse* garnered a median of 4 (IQR 3-9) upvotes and 4 (IQR 2-5.5) comments.

## Discussion

### Overview

Using data from all quit vaping subreddits, this study shows that post volume has been increasing over time. This finding emphasizes the increasing use of online forums for seeking quit vaping resources within the context of increasing population vape use. This work also identifies that people are using these forums to both request and provide social support from their peers. Broadly, these findings are further evidence that along with increasing vape product use, there is also an increasing desire to quit. Reddit forums are serving as a digital community where individuals gather to seek support to quit vaping and to provide quitting support to others. The presence of both support-seeking and support-providing behaviors suggests the forum encompasses variable types of engagement regarding the quitting process.

The increasing post volume on quit vaping subreddits reflects a growing public appetite for quitting resources as vaping continues to proliferate [1,2]. This increase in post volume also suggests a desire for the peer-to-peer social support offered by Reddit forums. We find this reflected in the post content, with 2 out of every 3 posts seeking or providing social support. It is also important to note that the posts *providing* social support garnered 3 times as many upvotes, that is, positive engagement, as the posts *seeking* social support, suggesting many Reddit users may be seeking out motivational support from their peers. Prior literature has established that peer-to-peer social support can be a valuable tool for tobacco cessation [14,25,36-38]. Furthermore, this type of peer support is replicated in the messaging design of successful text-to-quit vaping interventions [39] and is in line with prior literature around vaping cessation [25]. This analysis highlights the increasing desire for peer-to-peer social support in the quit vaping experience, outlining an important consideration for effective intervention implementation.

This study supports research suggesting that societal events shape individual quitting behaviors. While attribution of the observed patterns to discrete events is not possible with the current dataset, we offer the following speculative explanations based on temporally co-occurring events for investigation in future research. The sharp rise in post volume in the Fall of 2019 suggests that interest in quitting vaping spiked around the time when lung injury initially connected to vaping was making national headlines [40,41]. Similarly, we also note that, over time, the most trafficked subreddit changed from r/QuittingJuul to r/QuitVaping. This shift mirrors the market expansion that occurred after JUUL’s sale was heavily restricted and product users began picking up disposable products that continue to be sold in a variety of flavors [18,19]. Finally, we note that post volume did increase from 2020 onward, after the dawn of the COVID-19 pandemic. Studies show that while some increased their use of tobacco during the pandemic, others felt motivated to quit during this time, likely turning to online accessible quit vaping resources [42]. Although few implications can be drawn from these overlapping phenomena, further research should investigate the extent to which information-seeking behavior is responsive to tobacco control policy events and media coverage. This could encourage interventionists to utilize future policy and media events to their advantage, seeking out potential quitters at these shared moments.

When examining the content of the posts, our work builds on prior literature, finding that battling withdrawal is a significant barrier to quitting while improving health is a frequently listed motivator [29]. Coping with withdrawal and other common topics, including quit strategies and relapse, align with evidence-based suggested topics for discussion with tobacco users from the US Public Health Service Clinical Practice Guidelines for Treating Tobacco Use and Dependence [43] as well as previous findings from other digital cessation communities [44].

In addition to confirming these trends, we also note that users shared specific strategies for quitting vaping that were contextualized inside of an expanding nicotine market. Specifically, participants mentioned tapering down their nicotine content, as others have previously observed [29]. Some participants went so far as to provide extensive guides to this stepping-down process. This methodology is specific to quitting vaping but bears similarity to clinical guidance provided for tapering nicotine with FDA-approved NRT [43]. Additionally, when considering strategies for quitting, participants mentioned using not only NRT designed for smoking cessation but also products not approved for cessation, such as newly popular nicotine pouch products. More research is needed to identify how those seeking to quit vaping are viewing these products within the context of quitting or cutting back on nicotine, as compared to FDA-approved NRT.

These findings have direct implications for the development and implementation of quit vaping resources. The characteristics of the Reddit forum itself could be indicative of what individuals might seek out from a quit vaping resource, for example, information sharing and social support. The content also indicates that quit vaping support messaging should be tailored to the way these products are used in an expanding nicotine market, such as tapering down nicotine content or product substitution. Additionally, beyond lessons learned from the content itself, the growing presence on these subreddits indicates a growing population of individuals who want to quit vaping and are turning to social media sites for help. These individuals have created their own quitting support, and many could likely benefit from quit programs. Future research should investigate ties between quit program use and tobacco policy events.

### Limitations

This work has several limitations. Not all information was complete across post, comment, and user datasets as some posts or accounts had been removed by users. There is subjectivity in inductively creating and assigning codes for a dataset due to the inherent biases of those conducting the interpretation. The preliminary topic model was introduced to reduce the likelihood of these inherent biases affecting the interpretation of the data in the development of coded categories. Finally, we are attempting to understand a population based on a convenience sample; those participating in quit vaping subreddits may not be representative of all individuals wishing to quit vaping, nor all individuals participating in other digital cessation communities, which may have different norms. Specifically, Reddit users are disproportionately White young adult men, limiting the generalizability of the results to the whole population, as is typical of a convenience sample.

### Conclusions

We find an increasing desire for peer-to-peer support for quitting vaping over time on Reddit, indicating a greater need for quit vaping resources as vaping continues to gain popularity in the United States [1-3]. We also note that the content on the quit vaping subreddits affirms the sharing of barriers and facilitators to quitting identified by prior literature but also highlights important characteristics of quitting vaping specific to an expanding and changing nicotine product environment, providing critical guidance for the development of quit vaping tools.

## Abbreviations

FDA: Food and Drug Administration
LDA: latent Dirichlet allocation
NRT: nicotine replacement therapy
PMTA: premarket tobacco product application

## Data Availability

The datasets generated and analyzed during this study are available from the corresponding author on reasonable request.
